# *APOL1-G2* accelerates nephrocyte cell death by inhibiting the autophagy pathway

**DOI:** 10.1242/dmm.050223

**Published:** 2023-12-27

**Authors:** Jun-yi Zhu, Jin-Gu Lee, Yulong Fu, Joyce van de Leemput, Patricio E. Ray, Zhe Han

**Affiliations:** ^1^ Center for Precision Disease Modeling, Department of Medicine, University of Maryland School of Medicine, Baltimore, MD 21201, USA; ^2^ Division of Endocrinology, Diabetes and Nutrition, Department of Medicine, University of Maryland School of Medicine, Baltimore, MD 21201, USA; ^3^ Department of Pediatrics, Child Health Research Center, University of Virginia, Charlottesville, VA 22903, USA

**Keywords:** APOL1, Podocyte, Nephrocyte, *Drosophila*, Endocytosis, Autophagy

## Abstract

People of African ancestry who carry the *APOL1* risk alleles *G1* or *G2* are at high risk of developing kidney diseases through not fully understood mechanisms that impair the function of podocytes. It is also not clear whether the *APOL1-G1* and *APOL1-G2* risk alleles affect these cells through similar mechanisms. Previously, we have developed transgenic *Drosophila melanogaster* lines expressing either the human *APOL1* reference allele (*G0*) or *APOL1-G1* specifically in nephrocytes, the cells homologous to mammalian podocytes. We have found that nephrocytes that expressed the *APOL1-G1* risk allele display accelerated cell death, in a manner similar to that of cultured human podocytes and APOL1 transgenic mouse models. Here, to compare how the *APOL1-G1* and *APOL1-G2* risk alleles affect the structure and function of nephrocytes *in vivo*, we generated nephrocyte-specific transgenic flies that either expressed the *APOL1-G2* or both *G1* and *G2* (*G1G2*) risk alleles on the same allele. We found that *APOL1-G2-* and *APOL1-G1G2*-expressing nephrocytes developed more severe changes in autophagic pathways, acidification of organelles and the structure of the slit diaphragm, compared to *G1*-expressing nephrocytes, leading to their premature death. We conclude that both risk alleles affect similar key cell trafficking pathways, leading to reduced autophagy and suggesting new therapeutic targets to prevent APOL1 kidney diseases.

## Teaser

**Summary:** Nephrocyte-specific expression of *APOL1* reference (G0) or risk alleles (G1, G2, G1G2) in *Drosophila* revealed *APOL1-G2* to be most pathogenic in disrupting nephrocyte function and filtration structure, and implicates underlying dysfunctional autophagy.

## INTRODUCTION

Apolipoprotein L1 is encoded by the *APOL1* gene, which carries well-established risk variants associated with a wide spectrum of kidney-related diseases, including focal segmental glomerulosclerosis (FSGS), HIV-associated nephropathy (HIVAN) and hypertension-associated end-stage renal disease (ESRD) (Kopp et al., 2011; Tzur et al., 2012; Ulasi et al., 2013). It is unique to humans and some primates (Thomson et al., 2014). Circulating APOL1 protein forms part of the trypanolytic complexes that protect humans against infections with the *Trypanosoma brucei* (*T. brucei*) that cause African sleeping sickness. However, subspecies of *T. brucei* (including *T. brucei rhodesiense* and *T*. *brucei gambiense*) have developed resistance to the ancestral G0 allele of *APOL1*, giving rise to the expansion of G1 and G2 alleles in the African population as they confer greater protection against the new subspecies.

*APOL1-G0* is generally considered the reference allele; it encompasses all APOL1 variants except *APOL1-G1* or *APOL1-G2*, both of which are known as the risk alleles. APOL1-G1 is denoted by the presence of two amino acid substitutions (S352G and I384M), while APOL1-G2 is marked by deletion of two amino acids (del388N389Y) (Genovese et al., 2010; Tzur et al., 2010). African Americans show an allele frequency for APOL1-G1 and APOL1-G2 of 23% and 15%, respectively, making these alleles the most common and powerful risk variants identified to date (Friedman and Pollak, 2020). Overall, ∼13% of African Americans carry the *APOL1* high-risk genotype (two risk alleles) that confers a 3–30-fold increased risk of developing different types of kidney disease (Friedman and Pollak, 2020; Limou et al., 2014).

The *APOL1-G2* allele appears to be the evolutionary older variant, which arose in response to the emergence of the serum resistance-associated’ protein (SRA) of *T. brucei* (Vanhamme et al., 2003; Molina-Portela et al., 2008), which binds and neutralizes APOL1-G0. *In vitro* studies have shown that APOL1-G2 exhibits almost no binding to SRA (Thomson et al., 2014); therefore, APOL1-G2 can render the parasite inactive, ensuring that humans remain safeguarded against infection (Beckerman and Susztak, 2018; Cooper et al., 2017). In contrast, APOL1-G1, has reduced binding to APOL1 compared to that of APOL1-G0, and appeared later in response to infections with *T. brucei gambiense*, which also evolved other mechanisms to block the trypanosomal effects of APOL1-G0 (Cooper et al., 2017; Friedman and Pollak, 2020; Thomson et al., 2014). The APOL1-G1 variant does not prevent infection with *T. brucei gambiense*, but reduces the symptoms of African sleeping sickness. In contrast, APOL1-G2 increases the risk of severe disease from a *T. brucei gambiense* infection (Cooper et al., 2017; Friedman and Pollak, 2020). These significant protective differences between APOL1-G1 and APOL1-G2 raise the question whether the two gene variants may also play different roles in the pathogenesis of chronic kidney disease. This notion is supported by findings in a cohort of sickle cell disease patients of Sub-Saharan African ancestry, which found that people carrying the *APOL1-G2* risk allele were more likely to have more-severe nephropathy than those carrying the *APOL1-G1* risk allele (Kormann et al., 2017). In addition, other differences were observed between the *G1* and *G2 APOL1* kidney risk alleles. In particular, *APOL1-G2* does not appear to increase the risk of heterozygous individuals to develop ESRD) or HIVAN, or the age in which the kidney diseases appeared but, unlike the *G1* allele, is present in all documented individuals with interferon-associated APOL1 nephropathy (Friedman and Pollak, 2020; Nichols et al., 2015). Whether the APOL1 risk variants G1 and G2 differ in their biologic behavior to trigger APOL1 kidney diseases remains unclear.

A recent study in fly garland cells – which carry out a filtration function similar to that of podocytes – has shown that both *G1* and *G2* alleles induce cytotoxicity when expressed, and implicated ER stress as the causal mechanism (Gerstner et al., 2022). However, technical limitations did neither allow the detection of acidification or functional uptake deficits, nor the distinction between APOL1-G1 and APOL1-G2 toxicity. Therefore, to determine whether the *APOL1-G1* and -*G2* alleles play similar or different roles precipitating APOL1 kidney diseases, we expanded the scope of our previous fly study (Fu et al., 2017a) by generating flies that selectively express the *APOL1* nephropathy risk alleles *G2* or *G1G2* (the latter comprising both mutations in one allele), and comparing the results with those generated in control (hereafter referred to as *Dot*-Gal4), *APOL1-G0* and *APOL1-G1* transgenic flies. Although no patients are known to carry the *APOL1-G1* and *APOL1-G2* mutations in the same allele, we included the APOL1-G1G2 variant to study whether the APOL1-G1 and APOL1-G2 risk variants act synergistically or independently, or are able to reduce detrimental effects. We used flies, a low cost and a highly efficient screening system, because our previous findings by using this model have since been validated and expanded by other groups in yeast, cultured human podocytes and *APOL1* transgenic mice (Beckerman et al., 2017; Fu et al., 2017a; Kruzel-Davila et al., 2017; McCarthy et al., 2021). We found that overexpression of *APOL1-G1* and *APOL1-G2* risk alleles – specifically in the fly's nephrocytes, the equivalent of mammalian podocytes – led to deficits in nephrocyte function, decreased cell numbers, impaired organelle acidification, and a worsening phenotype with age. In agreement with previous studies done in *APOL1* transgenic mice (McCarthy et al., 2021), we found that the *APOL1-G2* risk allele induced a more severe phenotype when compared to *APOL1-G1*. We further gained mechanistic insights by showing that *G1* and *G2* risk allele phenotypes are marked by disrupted nephrocyte slit diaphragm structures as well as altered endocytic membrane trafficking and autophagy pathways.

## RESULTS

### Generation of fly models for human APOL1-G2 and APOL1-G1G2 allelic variants

**Fig. 1.**
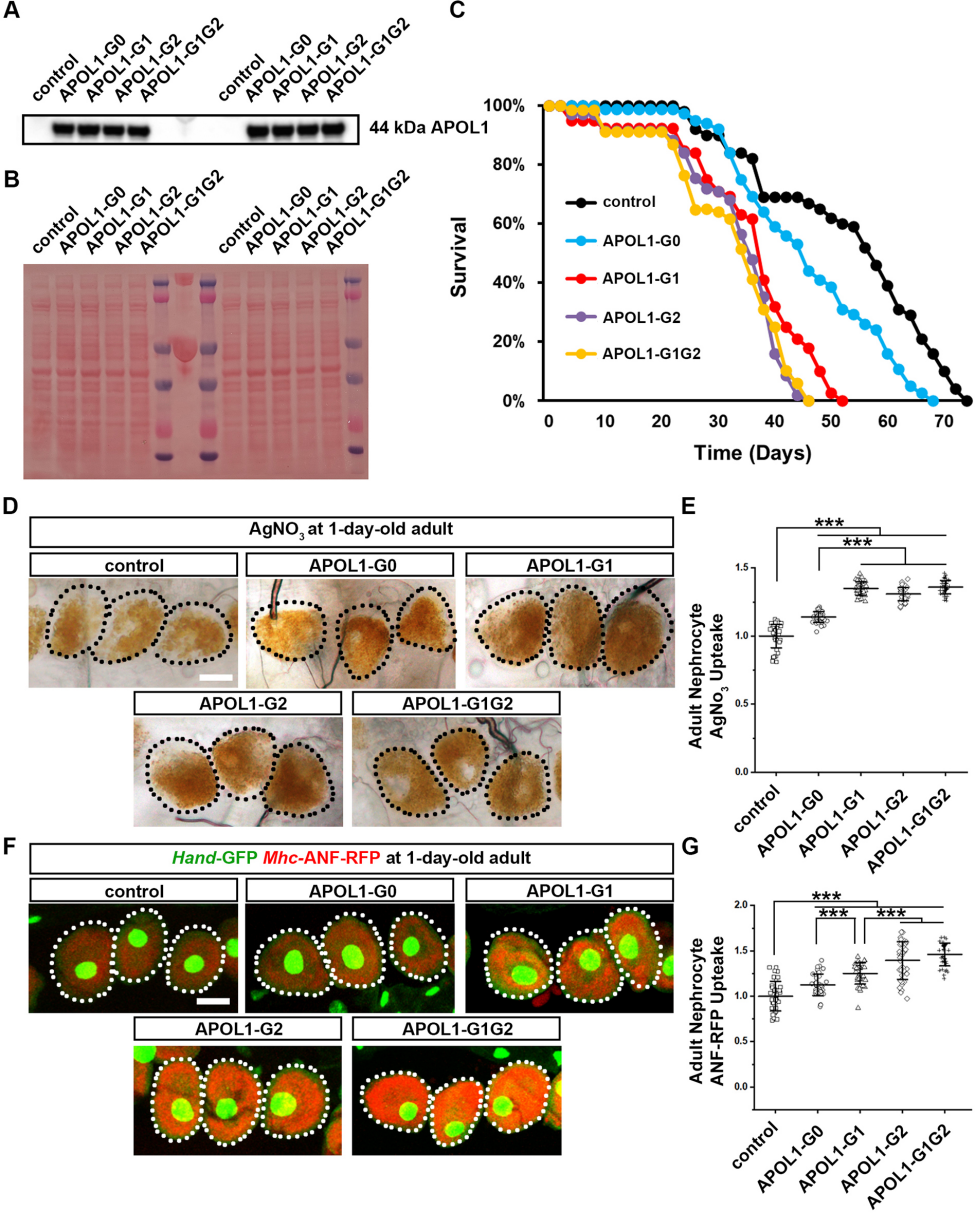
**Expression of *APOL1-G2* or *APOL1-G1G2* increases nephrocyte function in 1-day-old adult flies compared to expression of *APOL1-G1*.** (A) Western blot analysis of APOL1 protein (44 kDa) expression using APOL1 antibody for control (*tub*-Gal4) and each of the APOL1 constructs. (B) Total protein loaded on western blot in A. Total protein was extracted from 3rd instar larvae carrying *tub*-Gal4 to drive *APOL1* transgene expression and control larvae carrying *tub*-Gal4 only. (C) Plotted is the survival over time for control and *Dot* enhancer-directed *APOL1* transgene-expressing adult flies. *n*=100 flies per group. (D) Silver nitrate (AgNO_3_) uptake by nephrocytes of a 1-day-old adult fly using the nephrocyte-specific driver *Dot*-Gal4 to express *APOL1-G0*, *APOL1-G1*, *APOL1-G2* or *APOL1-G1G2* alleles. Dotted lines outline nephrocytes. Scale bar: 25 µm. (E) Quantification of AgNO_3_ uptake by *APOL1* allelic variant nephrocytes, relative to control nephrocyte function. *n*=40 nephrocytes from six flies per group. (F) Uptake of hemolymph proteins by nephrocytes. *Mhc*-ANF-RFP uptake (red) by nephrocytes from 1-day-old adult flies using the nephrocyte-specific driver *Dot*-Gal4 to express *APOL1-G0*, *APOL1-G1*, *APOL1-G2* or *APOL1-G1G2* alleles. *Hand*-GFP transgene expression (green) concentrated in the nuclei of nephrocytes. Dotted lines outline the nephrocytes. Scale bar: 25 µm. (G) Quantification of ANF-RFP uptake by APOL1 allelic variant nephrocytes, relative to control nephrocyte function. *n*=40 nephrocytes from six flies per group. Results are presented as the mean±s.d. Kruskal–Wallis H-test followed by a Dunn's test; statistical significance: ***P<0.001.

Since fly does not carry a gene homologous to *APOL1*, we produced transgenic *Drosophila melanogaster* lines that carry the human *APOL1-G0* reference allele (obtained from OriGene, with M228 and R255 mutagenized to I228 and K255 to match APOL1-G1) or an *APOL1-G1* risk allele that had been derived previously from a patient diagnosed with HIVAN (Fu et al., 2017a). Here, we expanded on this fly model by generating *APOL1-G2* and *APOL1-G1G2* transgenic fly lines. The *APOL1-G2* risk allele carries two amino acid deletion points (at N388del and Y389del) and the *APOL1-G1G2* risk allele carries both the *APOL1-G1* point mutations (S342G and I384M) and the *APOL1-G2* deletion mutations (N388del and Y389del). To ensure equal expression of all constructs we carried out western blot for APOL1 in flies that expressed the APOL1-G0 or APOL1-G1 [previous constructs; *P* element insertion (Fu et al., 2017a)], or G2 or APOL1-G1G2 (fixed docking site). We used the ubiquitous *tubulin* (*tub*; *alphaTub84B*) to drive expression (*tub*-Gal4; BDSC 30029) for the western blots as nephrocytes by themselves (*Dot*-Gal4 driver) did not yield sufficient material. Data showed equal expression for all *APOL1* alleles in the fly larvae (Fig. 1A,B). These newly generated fly models for *APOL1*-dependent risk for susceptibility to renal disease were used in all assays described in this manuscript.

### Flies with nephrocyte-specific expression of *APOL1-G2* or *APOL1-G1G2* and, to a lesser extent, *APOL1-G1* demonstrated reduced lifespan

We have previously shown that *APOL1* risk allele-induced perturbation of nephrocyte function affected adult fly longevity (Fu et al., 2017a). That study was carried out at 29°C, the temperature at which Gal4 is most stable. In our current study, we reduced the temperature to 25°C to maintain the flies, under which Gal4 is less stable. This reduced APOL1 expression and, thus, APOL1-associated toxicity (including that of APOL1-G0). By reducing baseline toxicity, we aimed to increase the sensitivity to assays to detect differences between APOL1-G0, APOL1-G1 and APOL1-G2. Under these new conditions APOL1-G0 flies survived ∼10 days longer. However, they still had slightly reduced lifespans compared to control flies (*Dot*-Gal4), which survived ∼60 days (50%) (Fig. 1C). Flies with nephrocyte-specific expression of human *APOL1-G1*, *APOL1-G2* or *APOL1-G1G2* alleles displayed reduced lifespans, such that most flies (50%) survived fewer than 40 days and virtually none made it past 45 days (Fig. 1C). These data support that *APOL1* risk allele-induced disruption of nephrocyte function affects longevity in flies.

### *APOL1-G2* and *APOL1-G1G2* expression in 1-day-old adult fly nephrocytes led to increased uptake function, more so than *APOL1-G1*

To measure functional changes induced by nephrocyte-specific *APOL1* expression, we exploited the ability of nephrocytes to take up and sequester toxins. Ingested silver nitrate (AgNO_3_) showed normal levels of sequestration in nephrocytes of 1-day-old adult control flies (Fig. 1D,E). The amounts of AgNO_3_ sequestered in nephrocytes of *APOL1-G1*-, *APOL1-G2*- and *APOL1-G1G2*-expressing 1-day-old adult flies were significantly increased (∼35%) compared to that in nephrocytes of control flies (i.e. control nephrocytes) (Fig. 1D,E). Nephrocytes expressing *APOL1-G0* showed only moderately increased AgNO_3_ uptake compared to control nephrocytes (∼10%; *P*=0.004) (Fig. 1D,E). This finding suggests that expression of *APOL1* risk alleles in nephrocytes, leads to increased endocytosis.

Since AgNO_3_ uptake by nephrocytes is cumulative, it might mask any subtle differences conferred by the different risk alleles. To further assess the effects of *APOL1* on nephrocyte function, we employed a complementary assay that measures uptake of hemolymph proteins by the nephrocytes. A myosin heavy chain (*Mhc*) promoter-driven atrial natriuretic factor (ANF)–red fluorescent protein (RFP) (*Mhc*-ANF-RFP) transgene was introduced to the model flies expressing human *APOL1*. The *Mhc* promoter directs expression of the ANF-RFP fusion protein in muscle cells, which is secreted into the hemolymph (fly blood system). ANF-RFP is then removed from circulation through endocytosis by the nephrocytes. Quantification is based on RFP signal intensity in the nephrocytes, as captured by imaging. We found that nephrocytes carrying *APOL1-G2* or *APOL1-G1G2* alleles showed significantly increased RFP levels compared to control nephrocytes (∼40%), and displayed homogenous RFP distribution throughout the nephrocyte cytoplasm (Fig. 1F,G). Nephrocytes expressing *APOL1-G0* or *APOL1-G1* showed only a moderate increase in RFP levels compared to those of control nephrocytes (∼20%) (Fig. 1F,G). These data confirmed the increased endocytosis seen in APOL1 risk allele-expressing nephrocytes in the AgNO_3_ uptake assay and suggest this phenotype is stronger in nephrocytes carrying the *APOL1-G2* and *APOL1-G1G2* alleles compared to those carrying *APOL1-G1*. We postulate, the increased ANF-RFP signal is the result of reduced protein degradation in addition to increased endocytosis activity. If so, this could indicate altered organelle acidification as another consequence of *APOL1* risk allele expression.

### Flies expressing an *APOL1* risk allele in their nephrocytes showed age-related changes in function

**Fig. 2.**
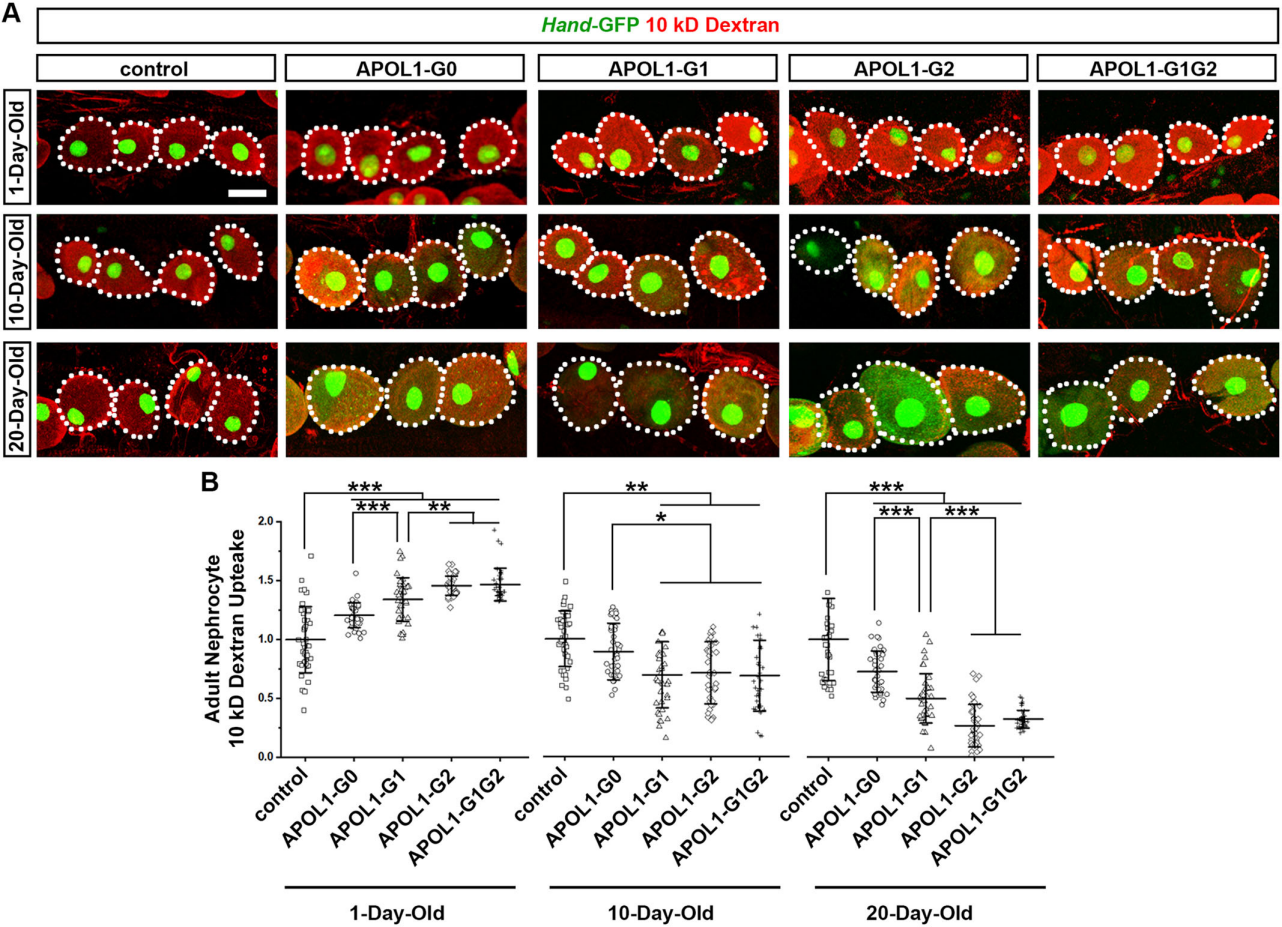
**Expression of *APOL1-G2* or *APOL1-G1G2* induces a more severe age-related phenotypic decline in nephrocyte than that of *APOL1-G1*.** (A) 10 kD fluorescent dextran particle uptake (red) by nephrocytes of 1-, 10- and 20-day-old adult flies by using the nephrocyte-specific driver *Dot*-Gal4 to express *APOL1-G0*, *APOL1-G1*, *APOL1-G2* or *APOL1-G1G2* alleles. *Hand*-GFP transgene expression (green) concentrated in the nuclei of nephrocytes. Dotted lines outline the nephrocytes. Scale bar: 40 µm. (B) Quantification of 10 kD dextran uptake by APOL1 allelic variant nephrocytes, relative to control nephrocyte function. *n*=40 nephrocytes from six flies per group. Results are presented as the mean±s.d. Kruskal–Wallis H-test followed by a Dunn's test; statistical significance: *P<0.05, **P<0.01, ***P<0.001.

*APOL1* risk allele-induced kidney function presents as age-related decline in human patients (Hoy et al., 2015). Therefore, we next tested if the nephrocyte functional phenotype in flies that express *APOL1* risk alleles shows a similar age-related pattern. Unfortunately, *Mhc*, which drives ANF-RFP expression in our functional assay, is not active in the skeletal muscle cells of adult flies. Therefore, we used an *ex vivo* functional assay instead, in which fly nephrocytes were dissected and then assayed for their capacity to filter and endocytose 10 kD dextran fluorescent particles from artificial hemolymph. Consistent with data from the AgNO_3_ and ANF-RFP assays, the 10 kD dextran *ex vivo* assay showed increased function of nephrocytes obtained from 1-day-old adult flies expressing an *APOL1* risk allele (Fig. 2A,B). Nephrocytes from *APOL1-G0* and *APOL1-G1* flies showed moderate fluorescent dextran accumulation compared to those from control animals (∼25% and 30%, respectively), while *APOL1-G2-* and *APOL1-G1G2*-expressing nephrocytes showed significantly greater accumulation of fluorescent dextran (∼40%) (Fig. 2A,B). Notably, nephrocytes obtained from 10- or 20-day-old flies expressing *APOL1* risk alleles showed a significant reduction in fluorescent dextran, indicating decreased nephrocyte function as opposed to the increased activity seen in nephrocytes obtained from 1-day-old adult flies. Nephrocytes from 10-day-old flies that expressed *APOL1-G1*, *APOL1-G2* or *APOL1-G1G2* showed a significant reduction in fluorescent dextran compared to those from control flies (∼40%) (Fig. 2A,B). Nephrocytes from 20-day-old flies displayed an even more severe phenotype, with those expressing *APOL1-G2* or *APOL1-G1G2* displaying ∼75% reduction in function compared to those of control flies. By contrast, nephrocytes expressing *APOL1-G0* or *APOL1-G1* showed a more moderate reduction (∼25% and 50%, respectively) (Fig. 2A,B). Together, these findings showed that flies carrying an *APOL1* risk allele exhibit age-related changes in nephrocyte function.

### Nephrocyte-specific expression of *APOL1* led to impaired endocytic membrane trafficking and disrupted autophagy

**Fig. 3.**
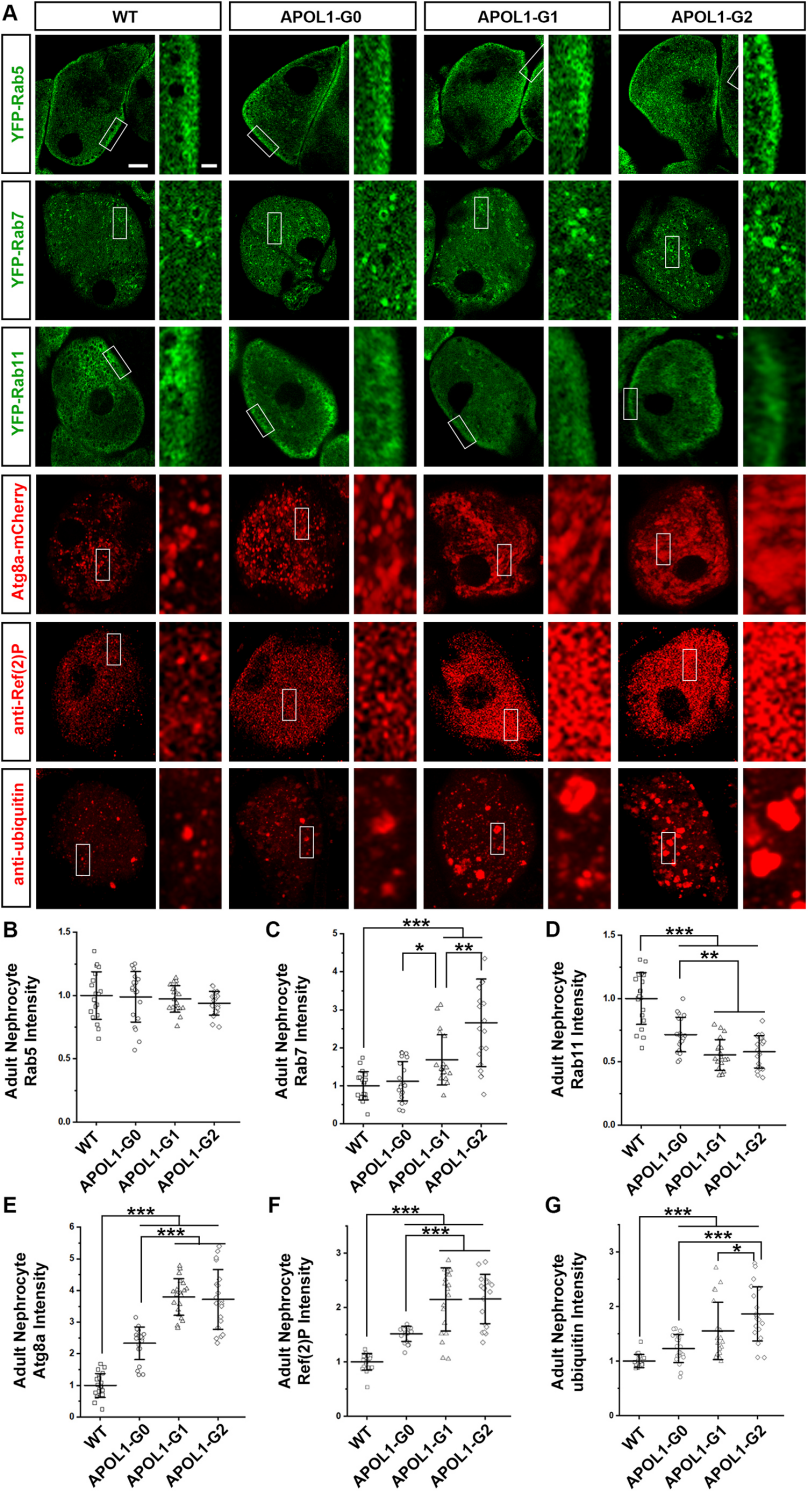
**Expression of *APOL1-G1* or *APOL1-G2* in nephrocytes leads to impaired endocytic membrane trafficking and induced autophagy.** (A) Expression of endocytosis-related proteins Rab5 (green, early endosome), Rab7 (green, late endosome), Rab11 (green, recycling endosome), autophagy related protein Atg8a (mCherry-Atg8a, red), autophagy receptor Ref(2)P [anti-Ref(2)P, red] and ubiquitinylated proteins (anti-ubiquitin, red) in nephrocytes of 1-day-old adult flies. The nephrocyte-specific driver *Dot*-Gal4 was used to express *APOL1-G0*, *APOL1-G1*, *APOL1-G2* or *APOL1-G1G2* alleles. Scale bar: 5 µm. Boxed areas are shown magnified to the right (scale bar: 1 µm). (B) Quantification of Rab5 fluorescence intensity in APOL1 allelic variant nephrocytes relative to control. *n*=20 nephrocytes from six flies per group. (C) Quantification of Rab7 fluorescence intensity in APOL1 allelic variant nephrocytes relative to control. *n*=20 nephrocytes from six flies per group. (D) Quantification of Rab11 fluorescence intensity in APOL1 allelic variant nephrocytes relative to control. *n*=20 nephrocytes from six flies per group. (E) Quantification of Atg8a fluorescence intensity in APOL1 allelic variant nephrocytes relative to control. *n*=20 nephrocytes from six flies per group. (F) Quantification of Ref(2)P fluorescence intensity in APOL1 allelic variant nephrocytes relative to control. *n*=20 nephrocytes from six flies per group. (G) Quantification of ubiquitinylated protein fluorescence intensity in APOL1 allelic variant nephrocytes relative to control. *n*=20 nephrocytes from six flies per group. Results are presented as the mean±s.d. Kruskal–Wallis H-test followed by a Dunn's test; statistical significance:*P<0.05, **P<0.01, ***P<0.001.

Next, we wanted to delve deeper into the changes that contribute to the nephrocyte dysfunctional uptake induced by the *APOL1* risk alleles. Therefore, we examined whether *APOL1* expression affects endocytosis by studying the localization of early endosome marker Rab5, late endosome marker Rab7 and recycling endosome marker Rab11. We chose these Rab proteins, as we had previously identified them as most important among the Rab GTPases expressed in and required for nephrocyte function (Fu et al., 2017b). We found expression of *APOL1-G0*, *APOL1-G1* or *APOL1-G2* in nephrocyte did not cause any changes in early endosomes (Rab5) but was associated with a significant reduction in recycling endosomes (Rab11) when compared to 1-day-old control flies. This phenotype was even more pronounced in nephrocytes expressing *APOL1-G1* or *APOL1-G2* (∼50%) (Fig. 3A,B,D). Notably, we did observe a significant increase (∼50%) in late endosomes (Rab7) within nephrocytes of 1-day-old flies expressing *APOL1-G1* compared to those of control flies (∼50%). This change was even greater (∼150%) in nephrocytes expressing *APOL1-G2* (Fig. 3A,C).

Furthermore, we studied the expression of autophagy- and degradation-related markers, including autophagosome marker autophagy-related 8a (Atg8a, known as GABARAP in human) and autophagy receptor refractory to sigma P [Ref(2)P, also known as p62, and SQSTM1 in human] as well as ubiquitinylated proteins marked for degradation (by using the antibody against ubiquitinylated proteins antibody clone FK2). We found a dramatic increase of autophagosome (∼250%, Atg8a) and autophagy receptor [Ref(2)P; ∼100%] in nephrocytes of 1-day-old flies expressing *APOL1-G1* or *APOL1-G2*, which was also significantly greater than that observed in *APOL1-G0* expressing nephrocytes (Fig. 3A,E,F). We also observed an ∼50% increase in the accumulation of ubiquitinylated proteins in nephrocytes expressing *APOL1-G0* or *APOL1-G1* compared to that in 1-day-old control flies, and this change was even greater (∼70%) in nephrocytes expressing *APOL1-G2* (Fig. 3A,G). These findings demonstrated that disrupted endocytosis mechanisms affecting the fusion of lysosomes and autophagosomes to inhibit autophagy pathways might contribute to the aberrant uptake function observed in nephrocytes expressing *APOL1*, especially its risk alleles.

### Nephrocyte-specific expression of risk alleles *APOL1-G2* or *APOL1-G1G2* rapidly and dramatically impairs acidification of organelles – more so than *APOL1-G1*

**Fig. 4.**
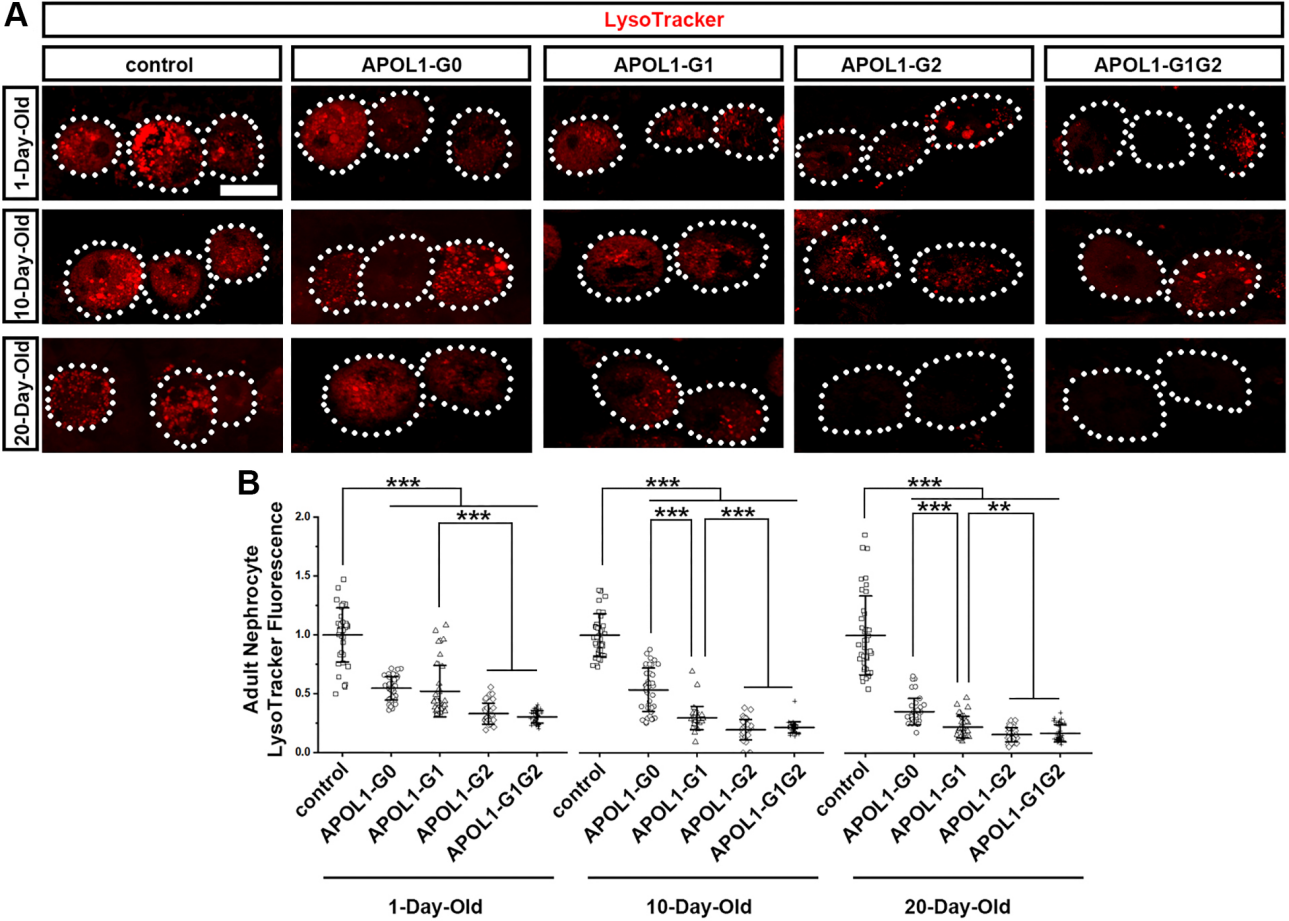
**Nephrocyte-specific expression of *APOL1-G2*, *APOL1-G1G2* or – to a lesser extend – *APOL1-G1* impairs the acidification of organelles.** (A) Fluorescence levels of LysoTracker dye (red) in nephrocytes of 1-, 10- and 20-day-old adult flies that express *APOL1-G0*, *APOL1-G1*, *APOL1-G2* or *APOL1-G1G2* alleles through the nephrocyte-specific driver *Dot*-Gal4. Dotted lines outline the nephrocytes. Scale bar: 40 µm. (B) Quantification of LysoTracker dye fluorescence intensity in APOL1 allelic variant nephrocytes relative to levels in control nephrocytes. *n*=40 nephrocytes from six flies per group. Results are presented as the mean±s.d. Kruskal–Wallis H-test followed by a Dunn's test; statistical significance: **P<0.01. ***P<0.001.

We have previously shown that ubiquitous *APOL1* transgene (*G0* or *G1* allele) expression leads to impaired organelle acidification in fly nephrocytes (Fu et al., 2017a). Thus, next we used fluorescent LysoTracker dye to examine the status of acidic vacuoles in flies with nephrocyte-specific expression of *APOL1* allelic variants. We found that, in nephrocytes of 1-day-old flies, expression of *APOL1-G0* or *APOL1-G1* was associated with a significant reduction in LysoTracker fluorescence (∼50%) compared to that in control flies. This reduction was even more pronounced (∼70%) in fly nephrocytes expressing *APOL1-G2* or *APOL1-G1G2* (Fig. 4A,B). The reduction in LysoTracker fluorescence –indicative of aberrant organelle acidification – declined further with age, as evident in 10- and 20-day-old flies displaying dramatically reduced levels in their nephrocytes (i.e. ∼75%, ∼80% or ∼80% in flies expressing *APOL1-G1*, *APOL1-G2* or *APOL1-G1G2*, respectively) compared to levels in nephrocytes of control flies (Fig. 4A,B). Nephrocytes expressing *APOL1-G0* showed a significant reduction in LysoTracker fluorescence compared to control nephrocytes at day 20 (∼65%) but not at day 10 (∼45%), when compared to levels at day 1 (∼45% reduction compared to control at this timepoint) (Fig. 4A,B). These data show that nephrocyte-specific expression of any *APOL1* allele resulted in impaired organelle acidification, and that this phenotype is most pronounced in flies expressing the risk alleles, especially *APOL1-G2* and *APOL1-G1G2*.

### *APOL1* expression in nephrocytes severely disrupted localization of Pyd, indicative of slit diaphragm structural defects in *Drosophila*

**Fig. 5.**
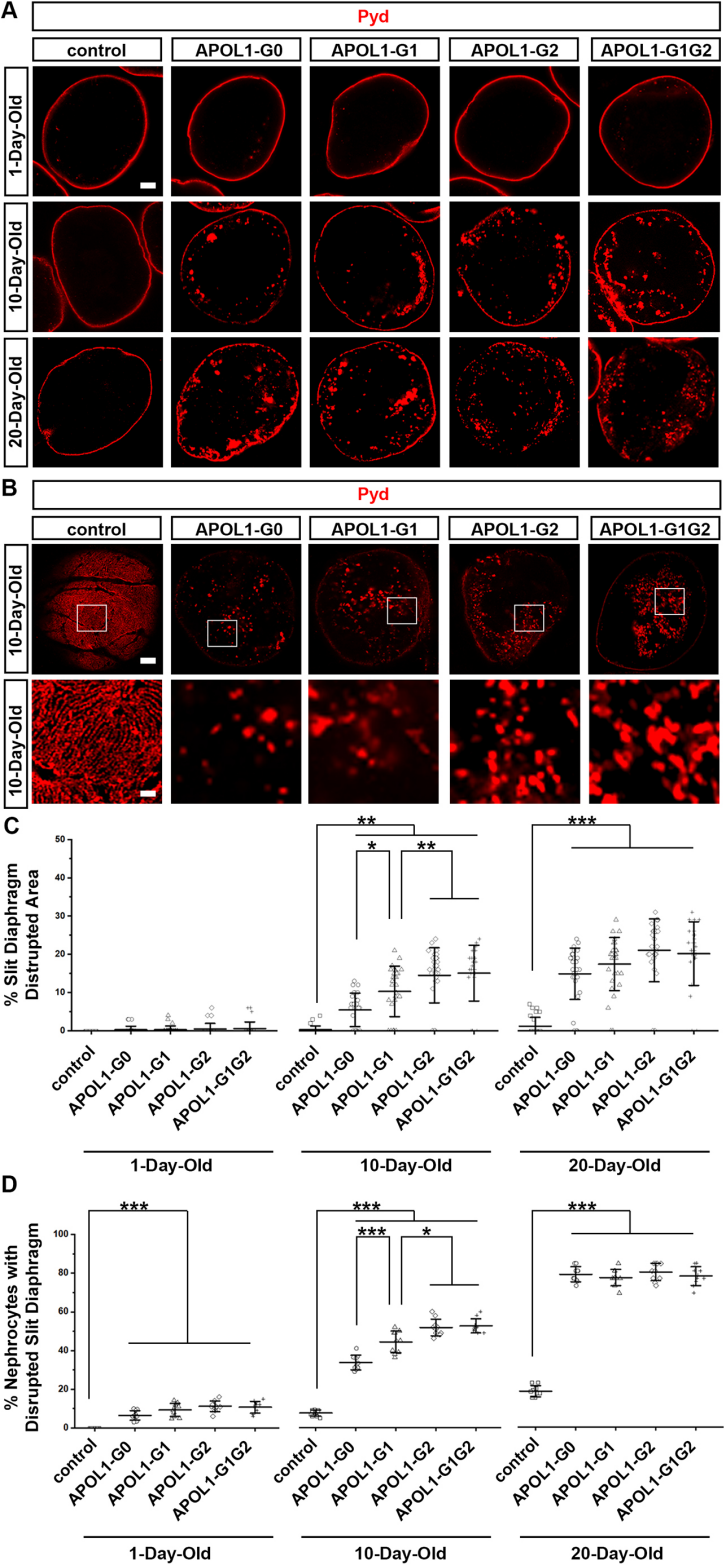
**Expression of *APOL1-G2*, *APOL1-G1G2* or *APOL1-G1* in nephrocyte disrupts the slit diaphragm structure.** Localization of slit diaphragm protein Pyd (red) by immunofluorescence in control and APOL1 allelic variant nephrocytes. *Dot*-Gal4 was used to drive nephrocyte-specific expression of *APOL1-G0*, *APOL1-G1*, *APOL1-G2* or *APOL1-G1G2* alleles. (A) Medial optical sections of nephrocytes obtained from 1-, 10- and 20-day-old adult flies. Scale bar: 5 µm. (B) Nephrocyte surface of 10-day-old adult flies. Scale bar: 5 µm. Boxed areas are shown magnified below each image. Scale bar: 1 µm. (C) Quantification of slit diaphragm distribution area (in %) based on the presence of intracellular Pyd in medial optical sections. *n*=30 nephrocytes from six flies per group. (D) Quantification of nephrocytes (in %) that displayed impaired slit diaphragm structure based on Pyd immunofluorescence. *n*=10 flies per group. Results are presented as the mean±s.d. Kruskal–Wallis H-test followed by Dunn's test; statistical significance: *P<0.05, **P<0.01, ***P<0.001.

Previously, we have shown that endocytosis-driven recycling is important for nephrocyte function and to maintain slit diaphragm structural integrity (Fu et al., 2017b; Wang et al., 2021; Wen et al., 2020). Since endocytosis is impaired in nephrocytes expressing an *APOL1* risk allele (Fig. 3), we had a closer look at the slit diaphragm, i.e. the filtration unit essential to nephrocyte functioning. To examine its structural integrity in nephrocytes expressing *APOL1*, we carried out immunochemistry for the slit diaphragm protein polychaetoid (Pyd) of *Drosophila*. Localization of Pyd in the medial optical section of nephrocytes was found to be a fine and continuously delineated circumferential ring in control flies (Fig. 5A). On the surface of control nephrocytes Pyd presented as a uniform and smoothly distributed finger-print-like localization pattern (Fig. 5B). Nephrocyte-specific expression of *APOL1* disrupted Pyd localization, such that Pyd protein was not longer at the surface but internalized. The vast majority of nephrocytes of 1-day-old flies appeared normal (∼10% show Pyd mislocalization) (Fig. 5D). However, with age, flies carrying any – i.e. non-risk or risk – *APOL1* allele showed increasingly disrupted Pyd surface localization pattern and increased Pyd internalization (Fig. 5A,C). This *APOL1*-induced phenotype is significantly more severe in nephrocytes of 10-day-old flies expressing alleles *APOL1-G2* or *APOL1-G1G2* (Fig. 5A-C), with signs of disrupted Pyd localization in *APOL1-G0* (∼30% of cells), *APOL1-G1* (∼40% of cells), *APOL1-G2* (∼50% of cells) and *APOL1-G1G2* (∼50% of cells) (Fig. 5D). This phenotype increasingly worsened with age, yet was comparable across all *APOL1* transgenic flies at 20-days-old (Fig. 5C), the age at which the vast majority of cells (∼80%) showed signs of a severely disrupted slit diaphragm structure (Fig. 5C,D). Taken together, these findings show that *APOL1*-induced impairment of nephrocyte function is probably due to structural disruption of the slit diaphragm, which is essential for filtration by the nephrocytes.

### Flies with nephrocyte-specific expression of *APOL1* risk alleles showed age-related increases in nephrocyte size and decreases in nephrocyte number

**Fig. 6.**
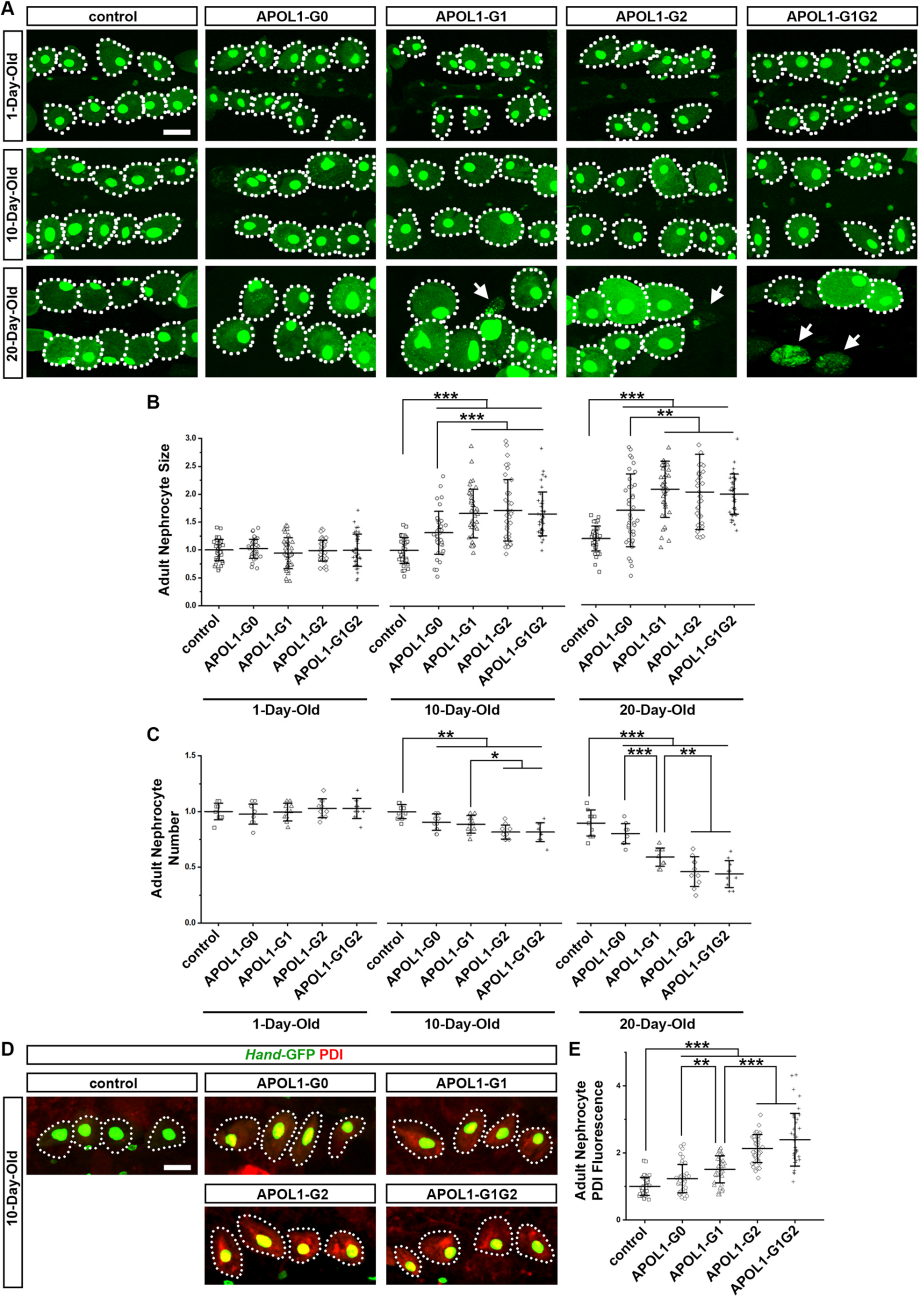
**Expression of *APOL1-G2* or *APOL1-G1G2* induces more severe age-related increases in nephrocytes size and reductions in nephrocyte number than those induced by *APOL1-G1*, with nephrocyte size increased and nephrocyte number decreased.** (A) Nephrocytes of 1-, 10- and 20-day-old adult flies expressing the APOL1 alleles *APOL1-G0*, *APOL1-G1*, *APOL1-G2* or *APOL1-G1G2* through the nephrocyte-specific driver *Dot*-Gal4. Expression of the *Hand*-GFP transgene (green) is concentrated in the nuclei of nephrocytes. Dotted lines outline the nephrocytes. Arrows point to debris visible near intact nephrocytes. Scale bar: 40 µm. (B) Quantification of nephrocyte size relative to that of control nephrocytes in adult flies at the indicated ages, expressing one of the four *APOL1* allelic variants. *n*=40 nephrocytes obtained from six flies per group. (C) Quantification of nephrocyte numbers relative to that of control nephrocytes in adult flies at the indicated ages, expressing one of the four *APOL1* allelic variants. *n*=10 flies per group. (D) Representative immunostaining images of nephrocytes for the endoplasmic reticulum (ER) stress marker protein disulfide isomerase (PDI; red) from 10-day-old adult flies. The nephrocyte-specific driver *Dot*-Gal4 was used to express *APOL1-G0*, *APOL1-G1*, *APOL1-G2* or *APOL1-G1G2* alleles. Expression of the *Hand*-GFP transgene (green) is concentrated within the nuclei of nephrocytes. Dotted lines outline the nephrocytes. Scale bar: 40 µm. (E) Quantification of PDI fluorescence intensity in APOL1 allelic variant nephrocytes as shown in D. *n*=40 nephrocytes obtained from six flies per group. Results are presented as the mean±s.d. Kruskal–Wallis H-test followed by Dunn's test; statistical significance: *P<0.05, **P<0.01, ***P<0.001.

We previously have shown that expression of *APOL1* transgenes (*APOL1-G0*, *APOL1-G1*) leads to an initial increase in cell function, followed by hypertrophy and, ultimately, cell death (Fu et al., 2017a). As this process is likely to relate to the age-related phenotypic decline observed in the *APOL1* risk allele flies in this current study, we examined the size and number of nephrocytes. Data showed that, as the flies aged, expression of an *APOL1* risk allele led to a significant increase in nephrocyte size (Fig. 6A,B). The 1-day-old flies did not show a difference in nephrocyte cell size regardless of their genotype. However, as the flies aged, those expressing *APOL1* in their nephrocytes displayed significantly increased cell sizes. In 10-day-old flies, nephrocytes expressing *APOL1-G0* displayed a moderate (∼20%) increase in cell size compared to control nephrocytes (Fig. 6B); however, those expressing any of the risk alleles (i.e. *APOL1-G1*, *APOL1-G2* or *APOL1-G1G2*) showed cell sizes significantly larger (∼50%) than control nephrocytes or *APOL1-G0*-expressing cells (Fig. 6B). Nephrocytes from 20-day-old flies showed an even greater increase in size, with cells expressing *APOL1-G1*, *APOL1-G2* or *APOL1-G1G2* being ∼80% larger than control nephrocytes (Fig. 6B).

In addition, expression of *APOL1* was associated with an age-related reduction in the number of nephrocytes (Fig. 6A,C). Moreover, we observed accumulation of cell debris in the vicinity of any remaining nephrocytes in flies expressing the *APOL1-G1*, *APOL1-G2* or *APOL1-G1G2* alleles (Fig. 6A, arrows). Overall, we found no difference in nephrocyte numbers in 1-day-old flies. However, in 10-day-old flies, expression of *APOL1-G0* or *APOL1-G1* led to a significant reduction (∼10%) in nephrocyte numbers (Fig. 6C), whereas flies expressing the *APOL1-G2* or *APOL1-G1G2* risk allele showed an even greater reduction (∼20%) in nephrocyte numbers compared to age-matched control flies (Fig. 6C). At 20 days, APOL1-G0 flies did not show a significant further reduction. However, nephrocyte numbers were significantly and more severely reduced in flies expressing *APOL1-G1* (∼40%) and, even more so, in those expressing *APOL1-G2* or *APOL1-G1G2* (both ∼60%) (Fig. 6C).

### *APOL1* risk alleles induced ER stress in nephrocytes

A previous study using 3rd instar larval garland cells, another type of nephrocyte in the fly, had revealed that APOL1 induces an endoplasmic reticulum (ER)-stress response (Gerstner et al., 2022). Therefore, we checked the level of protein disulfide-isomerase (PDI), a marker for ER stress. Compared with control flies, PDI levels in nephrocytes expressing *APOL1-G1*, *APOL1-G2* or *APOL1-G1G2* were increased in 10-day-old flies (Fig. 6D,E), the timepoint we first observed nephrocyte loss (Fig. 6C). In 10-day-old flies, expression of *APOL1-G1* led to a similar, significant increase in ER stress (∼50%) in the nephrocytes when compared to age-matched control flies (Fig. 6E). This increase in PDI levels was even greater in nephrocytes of flies expressing the *APOL1-G2* or *APOL1-G1G2* risk allele, showing ∼100% increase compared to control (Fig. 6E). Therefore, like in the larval garland cells used by Gerstner et al. (2022), APOL1 induced ER-stress in pericardial nephrocytes of adult flies.

## DISCUSSION

In our current study we developed new transgenic *Drosophila* lines expressing human *APOL1-G2* and *APOL1-G1G2* exclusively in nephrocytes. The *APOL1-G2* and *APOL1-G1G2* transgenic lines, as well as the *APOL1-G1* and *APOL1-G0* lines had been made previously (Fu et al., 2017a), and all used the same nephrocyte-specific Gal4 driver (*Dot*-Gal4). Despite different insertion sites, all four constructs yielded equal APOL1 expression, which is important as even APOL1-G0 reference allele overexpression is known to induce toxicity. Given that both unnatural and different haplotype backgrounds can alter APOL1 cytotoxicity (Lannon et al., 2019), our transgenic flies are based on *APOL1-G1* cDNA derived from cultured podocytes that had been obtained from a child diagnosed with HIVAN (Xie et al., 2014) as described before (Fu et al., 2017a). The *APOL1-G1* cDNA contains the haplotypes E150, I228 and K255, and differs from the *APOL1-G0* cDNA only at S342 and I384 (*APOL1-G0* was obtained from OriGene and mutagenized to match *APOL1-G1*; M228I and R255K). The *APOL1-G2* cDNA contains the *APOL1-G1* haplotypes E150, I228 and K255, as well as dual deletion of amino acids N388 and Y389, characteristic of APOL1-G2. Therefore, all our transgenic constructs are physiologically relevant and carry common haplotypes seen in people of African ancestry.

All fly lines expressing *APOL1*, including *APOL1-G0*, in their nephrocytes showed – as the flies aged – a decline of nephrocyte function, increase in cell size, reduction in acidification of organelles and premature death of nephrocyte. Whereas we had previously kept the flies at 29°C, the temperature at which Gal4 is most stable (Fu et al., 2017a), we reduced the temperature to 25°C for this current study. This renders Gal4 less stable, thus reducing APOL1 expression. By doing so, we aimed to reduce baseline toxicity and increase assay ability to detect changes between the different *APOL1*-alelles. APOL1-G0 flies showed longer survival curves at 25°C compared to our previous study. When comparing the nephrocyte phenotypes among the different risk alleles, the data predominantly demonstrated that APOL1-G2- and APOL1-G1G2-expressing nephrocytes displayed more severe structural and functional changes, relative to nephrocyte-specific transgenic flies expressing the *APOL1-G1* allele obtained from a child diagnosed with HIVAN and the minimized effects observed in APOL1-G0 nephrocytes. Although improved, our assays still show residual toxicity in APOL1-G0 nephrocytes and, therefore, we were unable to detect differences between APOL1-G1 and APOL1-G2 in all assessments, including autophagy markers. Therefore, additional assays that directly assess autophagy or a means to further reduce APOL1 baseline toxicity, are needed to tease out in which cases APOL1-G2 is more toxic than APOL1-G1, distinctions that could inform pathomechanistic insights. Additional informing would be to understand how APOL1-G2 induces a more severe phenotype than APOL1-G1. One possibility is a different intracellular localization of each. Unfortunately, technical limitations currently prevented us from providing direct evidence for this hypothesis.

Although, in humans, *APOL1-G1* and *APOL1-G2* are not expressed on the same allele, we still included this artificial variant APOL1-G1G2 to determine whether both risk variants act in a synergistic or independent manner, or whether one variant could minimize the detrimental effects of the other. We found that both variants act in a similar manner by disrupting key cell trafficking pathways and confirmed that these changes were driven initially by the increased endocytic activity of nephrocytes, leading to the accumulation of proteins. These changes were followed by the premature death of nephrocytes. Overall, the changes induced by the *APOL1-G1* and *APOL1-G2* alleles in fly nephrocytes show that the endocytic function is initially increased in association with perturbations in endosomal trafficking pathways and acidification processes that impair the autophagic flux and degradation mechanisms of these cells.

Fly nephrocytes are highly active in endocytosis, since they need to take up material from the hemolymph (the blood of the fly) and then sort the endocytic cargo for either degradation in the lysosome or recycling back to the hemolymph (Fu et al., 2017b; Wang et al., 2021; Wen et al., 2020). Rab GTPases play a critical role in endocytosis and cell trafficking, acting as switches that recruit effector molecules that regulate these processes. Previously, we have shown that Rab5, Rab7 and Rab11 play key roles in maintaining the normal function of fly nephrocytes (Fu et al., 2017b; Wang et al., 2021; Wen et al., 2020). Rab5 is localized to early endosomes and plays a critical role regulating the early steps of endocytosis in nephrocytes. However, we did not detect significant changes in the expression levels of Rab5 despite the enhanced endocytic function of nephrocytes detected in 1-day-old transgenic flies expressing the *APOL1* risk alleles (evident in increased AgNO_3_ and ANF-RFP uptake). Thus, the methods we used to detect changes in Rab5 expression might not be sensitive enough or, alternatively, other GTPases might regulate this process as well. However, we did find a significant upregulation of Rab7 expression in nephrocytes expressing the *APOL1* risk alleles. We have previously reported that silencing Rab7 in nephrocytes results in the accumulation of clear vacuoles and a reduced number of lysosomes, in association with disrupted cell trafficking and degradation processes (Fu et al., 2017b). Therefore, we speculate that the changes in Rab7 expression levels (Fig. 3) reflect the activation of compensatory mechanism to re-establish the normal function of late endosomes as well as protein degradation processes. By contrast, expression of Rab11 was significantly reduced in nephrocytes expressing the *APOL1* risk alleles. Rab11 regulates the recycling of endosomal compartments, as well as the exocytosis of recycling vesicles at the plasma membrane in mammalian cells (Takahashi et al., 2012). Our findings support the notion that the function of recycling endosomal compartments is affected as well. Finally, we found high levels of the autophagosome marker Atg8a and autophagy receptor Ref(2)P in nephrocytes expressing *APOL1* risk alleles. These findings support that autophagosomes accumulate whenever the autophagic flux is impaired. Taken together, our findings suggest that *APOL1* risk alleles impair the function of nephrocytes mainly by disrupting late endocytic pathways that regulate the fusion of late endosomes with autophagosomes or lysosomes, therefore impairing the autophagic flux as well as protein degradation processes. Interestingly, similar findings have been reported in cultured human podocytes and in transgenic mice expressing APOL1 exclusively in podocytes. (Beckerman et al., 2017). Briefly, in these studies, the *APOL1* risk alleles reduced the autophagic flux of podocytes and increased the steady-state level of autophagosomes by impairing the fusion of lysosomes as well as degradation of autophagic cargo.

**Fig. 7.**
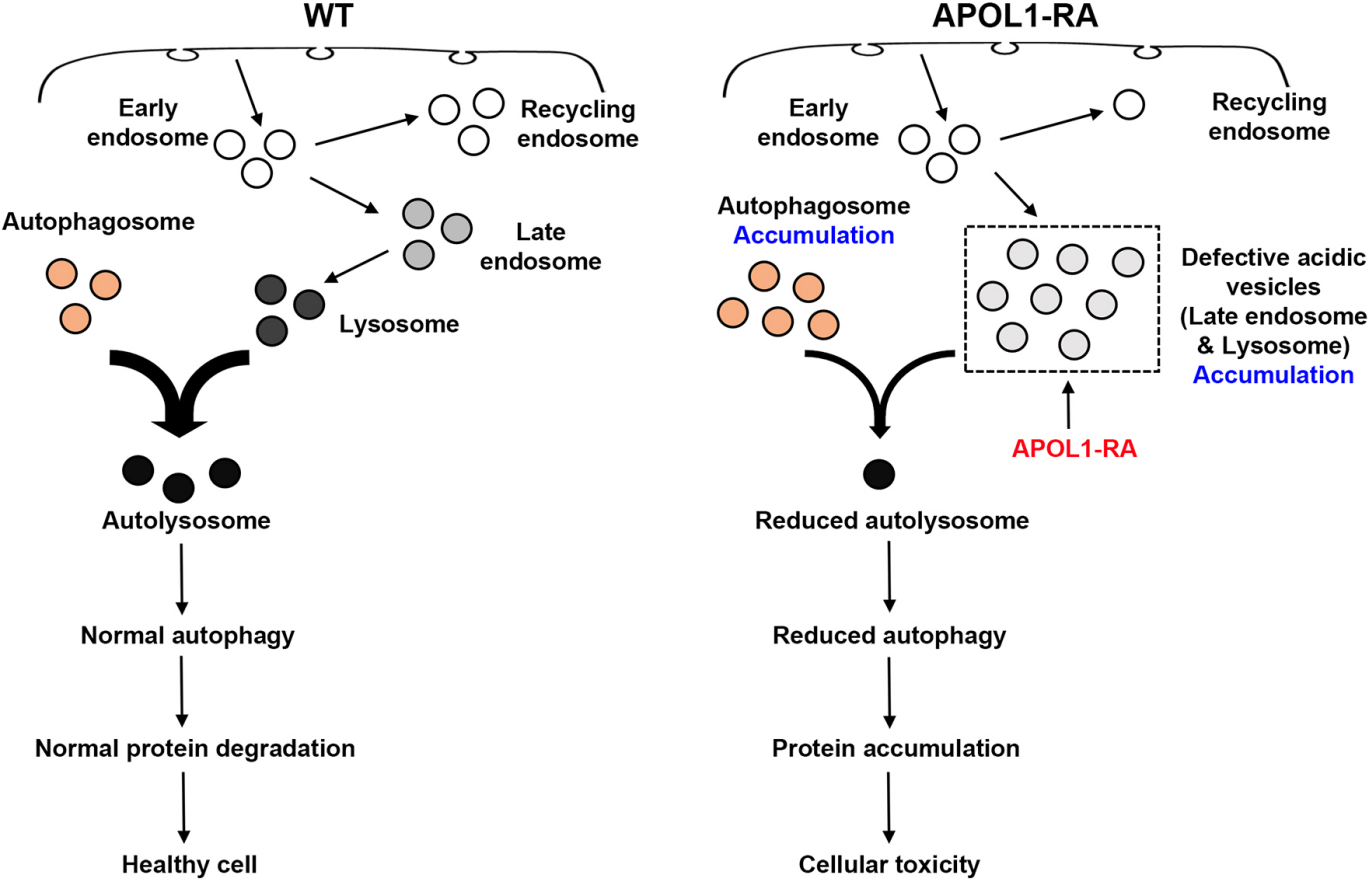
**Model of *APOL1* risk allele-mediated disruption of the autophagy pathway.** Graphic depiction of the proposed model by which *APOL1* risk alleles (APOL1-RA) disrupt an increased number of defective acidic vesicles, including late endosomes and lysosomes, thereby affecting the autophagy pathway by disrupting the fusion of autophagosomes and lysosomes, which – in turn – leads to protein accumulation and tissue damage.

The podocyte slit diaphragm is an extracellular structure that bridges the filtration slits between neighboring podocytes and is an essential component of the glomerular filtration barrier. Two of the three layers of the human glomerular filtration barrier, the slit diaphragm and the basement membrane, are present in fly nephrocytes. Here, we showed that the failure to maintain appropriate cell trafficking pathways affects the structure of the slit diaphragm and disrupts the process of endocytosis in nephrocytes expressing *APOL1* risk alleles (Fig. 7). Moreover, we reported that the presence of Pyd, a core component of the slit diaphragm, is greatly diminished and its characteristic fingerprint-like expression pattern is no longer visible (Fig. 5), indicating disrupted slit diaphragms as a plausible explanation for the reduced nephrocyte uptake function in older flies (Fig. 2). Disrupted slit diaphragm structures in humans are often indicative of dramatic podocyte morphological changes that lead to foot-process effacement associated with proteinuria, podocyte loss and, ultimately, kidney failure (Garg, 2018). Mice expressing the *APOL1-G1* or *APOL1-G2* risk allele in their podocytes display molecular, functional and structural podocyte changes, including foot-process effacement, that mimic the clinical features observed in patients with APOL1-induced kidney disease (Beckerman et al., 2017). Notably, the slit diaphragm proteins and their interactors, and the endocytosis and recycling pathway components, are highly conserved between fly and human (Wang et al., 2021). Overall, our findings are in line with previous studies carried out in yeast (Kruzel-Davila et al., 2017), cultured human podocytes, *APOL1* transgenic mice (Beckerman et al., 2017; McCarthy et al., 2021) and the age-related changes observed in patients with *APOL1*-associated kidney diseases (Abid et al., 2020; Hoy et al., 2015). However, the pathological changes induced by *APOL1* risk alleles in transgenic mice and cultured podocytes, appear to be regulated by the expression levels of these alleles and, in some transgenic mouse models – as it is the case in humans – a second ‘hit’ is needed, e.g. through high levels of interferon-γ (Aghajan et al., 2019; McCarthy et al., 2021) to induce the development of proteinuria and/or chronic kidney disease (Beckerman et al., 2017; Bruggeman et al., 2019; Okamoto et al., 2018; Ryu et al., 2019; Wakashin et al., 2020). One limitation of our fly model is that, given the high expression levels of APOL1 in the nephrocytes, a second hit is not needed to injure these cells.

The recent study in garland cells of *APOL1* transgenic flies, attributed the pathogenic effects of *APOL* risk alleles almost exclusively to ER stress (Gerstner et al., 2022). Gerstner and colleagues did not detect significant changes in cell trafficking pathways, organelle acidification or even the structure of the slit diaphragm. However, like us, they report that *APOL1* risk alleles increase the endocytic activity of nephrocytes and induce premature cell death (Gerstner et al., 2022); similar to their study, we detected a significant ER stress response. The different findings might be due to several factors: (i) garland cells and pericardial nephrocytes originate from different cell lineages, (ii) different overexpression constructs (e.g. drivers), (iii) different APOL1 background sequence and, (iv) different timepoints examined (3rd instar larva versus 1-, 10- and 20-day-old adult flies). Sufficient ER stress typically affects cell trafficking and degradation pathways; indeed, a study that used cultured human HEK293T cells found ER stress, endolysosomal disturbances and increased permeability of the cell membrane induced by *APOL* risk alleles (Granado et al., 2017). The authors suggested these to be secondary consequences of mitochondrial damage induced by APOL1 risk alleles. Since the garland cells showed ER stress in response to APOL1 and underwent premature cell death (Gerstner et al., 2022), perturbation of trafficking and degradation pathways would be expected, and additional studies are warranted.

In conclusion, we found that the *APOL1* risk alleles *G1* and *G2* affect similar key endocytic and cell trafficking pathways, and that the *G2* risk allele appears to induce more severe cytotoxic effects on nephrocytes than the *G1* risk allele. *Drosophila* pericardial nephrocytes provide a unique opportunity to assess the effects of *APOL1* risk alleles on different endocytic pathways *in vivo*, and to perform cost effective and large-scale genetic interactive screening to detect interacting molecules that affect endocytic activity and other cell trafficking pathways, and might be therapeutic targets to treat APOL1 kidney diseases. Previous studies in cultured human podocytes and *APOL1* transgenic mice support the main findings of our study. Nonetheless, it remains to be determined how these changes mimic those induced by *APOL1* risk alleles in patients with APOL1-associated kidney diseases.

## MATERIALS AND METHODS

### Fly strains

Flies were crossed, reared and kept on standard food (Meidi LLC) at 25°C. The following *Drosophila* strains used in this study had been previously generated by us (Z.H.’s lab): *Hand*-GFP (expressing green fluorescent protein in the nuclei of nephrocytes and cardiomyocytes; Han and Olson, 2005; Huang et al., 2023), and *Mhc*-ANF-RFP (expressing ANF red fluorescent protein in muscle myosin heavy chain promoter-driven atrial natriuretic peptide-red fluorescent protein in muscle cells; Zhang et al., 2013). The following lines were obtained from the Bloomington Drosophila Stock Center (BDSC; Indiana University, IN): *Dot*-Gal4 (ID 6903; *Ugt36A1* driver), *tub*-Gal4 (ID 30029; *alphaTub84B* driver), UAS-YFP-*Rab5* (ID 24616), UAS-YFP-*Rab7* (ID 23270), UAS-YFP-*Rab11* (ID 9790), and UAS-mCherry-*Atg8a* (ID 37750). As control, *w*^1118^ (BDSC; ID 3605) flies were used in the crosses.

### DNA cloning and generation of transgenic fly strains

The *APOL1-G0* and *APOL1-G1 Drosophila* strains had been generated by us (Z.H.’s lab) and are described elsewhere (Fu et al., 2017a). cDNAs of *APOL1-G0* and *APOL1-G1* alleles were cloned into the pUAST vector and introduced into the germ cells of flies by standard *P* element-mediated germ line transformation. The *APOL1-G2* and *G1G2* cDNAs were made using point mutagenesis and are based on the *APOL1-G1* cDNA (provided by P.E.R.) used previously to generate the *APOL1-G0* and *APOL1-G1* lines. This *APOL1-G1* cDNA was obtained from a patient of recent African ancestry. To generate *UAS-APOL1-G2* and *UAS-APOL1-G1G2* constructs, cDNAs of *APOL1-G2* and *APOL–G1G2* alleles were cloned into the pUASTattB vector, and the transgenes were introduced into a fixed chromosomal docking site by germ line transformation. To match the previous *APOL1-G0* and *APOL1-G1* constructs, we incorporated a FLAG epitope tag at the APOL1 protein C-terminus encoded by the *APOL1-G2* and *APOL1-G1G2* expression constructs, even though we did not need the FLAG-tag for the assays in this current study.

### Western blotting

3rd instar larvae from the *tub*-Gal4 transgenic lines were crossed to UAS-*APOL1-G0,* UAS-*APOL1-G1,* UAS-*APOL1-G2* or UAS-*APOL1-G1G2* transgenic lines. For each sample, five 3rd instar larvae were smashed in a 1.5-ml tube using a pestle. Immediately after, 500 µl RIPA lysis buffer with protease inhibitor was added, and tissue suspensions were sonicated, followed by centrifugation. The cleared tissue lysates were subjected to immunoblotting using anti-APOL1 (66124-1-Ig, Proteintech). Horseradish peroxidase (HRP)-conjugated secondary antibody (anti-mouse IgG-peroxidase antibody, Sigma #A4416; used at 1:2000 dilution) was used for detection, and the HRP signal was detected by using the enhanced chemiluminescence method (ECL) and recorded by using a G:BOX Chemi XRQ (Syngene).

### AgNO_3_ uptake assay

Female adult flies from the *Hand*-GFP and *Dot*-Gal4 transgenic lines were crossed to UAS-*APOL1-G0*, UAS-*APOL1-G1*, UAS-*APOL1-G2* or UAS-*APOL1-G1G2* transgenic lines. Eggs from these crosses were laid on standard food containing 0.002% AgNO_3_ (Sigma-Aldrich). AgNO_3_ uptake by nephrocytes was assessed *ex vivo* in 1-day-old adult flies by dissecting heart tissue into artificial hemolymph and examining the cells by phase-contrast microscopy after 10 min fixation in 4% paraformaldehyde in 1×phosphate buffered saline (1×PBS) (Thermo Fisher Scientific). For quantification, 40 nephrocytes were analyzed from six female flies in a single cross per genotype. The results are presented as the mean±standard deviation (s.d.). Statistical significance: *P<0.05, **P<0.01, ***P<0.001.

### ANF-RFP uptake assay

Female adult flies from the *Hand*-GFP, *Mhc*-ANF-RFP and *Dot*-Gal4 transgenic lines were crossed with flies from the UAS-*APOL1-G0*, UAS-*APOL1-G1*, UAS-*APOL1-G2* or UAS-*APOL1-G1G2* transgenic lines. Progeny from these crosses display nephrocyte-specific expression of one of the APOL1 allelic variants with nephrocytes labeled by GFP, as well as ANF-RFP expression driven by *Mhc*. RFP uptake by nephrocytes was assessed *ex vivo* in 1-day-old adult flies by dissecting heart tissue into artificial hemolymph and by examining cells by using fluorescence microscopy after 10 min fixation in 4% paraformaldehyde in 1×PBS (Thermo Fisher Scientific). For quantification, 40 nephrocytes from six female flies in a single cross per genotype were analyzed. The results are presented as mean±s.d. Statistical significance: *P<0.05, **P<0.01, ***P<0.001.

### Adult survival assay

Following egg laying, *Drosophila* larvae were kept at 25°C under standard conditions and at optimal temperature for UAS-*transgene* expression. Adult male flies were maintained in vials, in groups of 20 or fewer. The number of live flies in each group was recorded every second day. The assay was ended when no survivors were left for any of the lines. In total, 100 flies total from a single cross were assayed per genotype.

### Dextran uptake assay

Female adult flies from the *Hand*-GFP and *Dot*-Gal4 transgenic lines were crossed to UAS-*APOL1-G0*, UAS-*APOL1-G1*, UAS-*APOL1-G2* or UAS-*APOL1-G1G2* transgenic fly lines. Dextran uptake by nephrocytes was assessed *ex vivo* in adult flies by dissecting heart tissue into artificial hemolymph and examining cells by fluorescence microscopy following a 20 min incubation with Texas Red-dextran (10 kD, 0.05 mg ml^−1^, Invitrogen) and 10 min fixation in 4% paraformaldehyde in 1×PBS (Thermo Fisher Scientific). For quantification, 40 nephrocytes were analyzed from six female flies in a single cross per genotype. The results are presented as the mean±s.d. Statistical significance: *P<0.05, **P<0.01, ***P<0.001.

### LysoTracker assay

Adult flies from the *Hand*-GFP and *Dot*-Gal4 transgenic lines were crossed to UAS-*APOL1-G0*, UAS-*APOL1-G1*, UAS-*APOL1-G2* or UAS-*APOL1-G1G2* transgenic fly lines. LysoTracker intensity within nephrocytes was assessed *ex vivo* in adult flies by dissecting heart tissue into artificial hemolymph and examining cells by using fluorescence microscopy following 20 min incubation with LysoTracker (Red DND-99, Thermo Fisher Scientific) used according to the manufacturer’s instructions and 10 min fixation in 4% paraformaldehyde in 1×PBS (Thermo Fisher Scientific). For quantification, 40 nephrocytes were analyzed from six female flies in a single cross per genotype. The results are presented as the mean±s.d. Statistical significance: *P<0.05, **P<0.01, ***P<0.001.

### Immunochemistry

Adult female flies were dissected and heat-fixed for 20 s at 100°C in artificial hemolymph, i.e. 70 mmol/l NaCl (Carolina), 5 mmol/l KCl (Sigma), 1.5 mmol/l CaCl_2_·2H_2_O (Sigma-Alrich), 4 mmol/l MgCl_2_ (Sigma-Aldrich), 10 mmol/l NaHCO_3_ (Sigma-Aldrich), 5 mmol/l trehalose (Sigma), 115 mmol/l sucrose (Sigma-Aldrich), and 5 mmol/l HEPES (Sigma-Aldrich), in H_2_O. Primary mouse monoclonal anti-Pyd antibody (PYD2) was obtained from DSHB and used at a 1:100 dilution in 1×PBS with 0.1% Triton X-100 (Sigma) (PBST). Secondary antibody Alexa Fluor 555 (A-21422, Thermo Fisher Scientific) was used at a 1:1000 dilution in PBST. The nephrocytes were washed with PBST three times, blocked in PBST+2% bovine serum albumin (BSA; Sigma-Aldrich) for 40 min, incubated with primary antibodies at 4°C overnight, washed with PBST three times, incubated with secondary antibodies at room temperature for 2 h, washed with 1×PBST three times and mounted with Vectashield mounting medium (H-1000, Vector Laboratories).

Adult female flies were dissected and fixated for 10 min in 4% paraformaldehyde in 1×PBS (Thermo Fisher Scientific). Primary monoclonal mouse antibody ubiquitin FK2, raised against human polyubiquitin-B and polyubiquitin-C (BML-PW8810, Enzo Life Sciences) and used at a1:100 dilution in 1×PBS with 0.1% Triton X-100 (PBST, Sigma). Primary rabbit polyclonal antibody against Ref(2)P (ab178440, Abcam) was used at a 1:100 dilution in PBST. Primary rabbit polyclonal antibody against protein disulfide isomerase (PDI; P7122, Sigma) was used at a 1:100 dilution in PBST. Secondary antibody Alexa Fluor 555 (A-21422, Thermo Fisher Scientific) was used at a 1:1000 dilution in PBST. The nephrocytes were washed with PBST three times, blocked in PBST+2% BSA for 40 min, incubated with primary antibodies at 4°C overnight, washed with PBST three times, incubated with secondary antibodies at room temperature for 2 h, washed with 1×PBST three times and mounted with Vectashield mounting medium.

### Confocal imaging

Imaging for AgNO_3_ uptake was carried out with a Zeiss ApoTome.2 microscope using a 20× Plan-Apochromat 0.8 NA air objective. Confocal imaging for Dextran uptake, ANF-RFP, LysoTracker assays and anti-PDI was carried out using a Zeiss LSM900 microscope with a 20× Plan-Apochromat 0.8 NA air objective. Immunocytochemistry for anti-Pyd and anti-Ref(2)P were all carried out using confocal technology; a Zeiss LSM900 microscope with a 63× Plan-Apochromat 1.4 NA oil objective under Airyscan SR mode. Live-cell fluorescence imaging for the UAS-YFP-*Rab5*, UAS-*YFP-Rab7*, UAS-YFP-*Rab11*, and UAS-mCherry-*Atg8a* fly strains were all carried out using confocal technology; a Zeiss LSM900 microscope with a 63X Plan-Apochromat 1.4 NA oil objective under Airyscan SR mode. For quantitative comparison of intensities, common settings were chosen to avoid oversaturation. The results are presented as the mean±s.d. Statistical significance: *P<0.05, **P<0.01, ***P<0.001. ImageJ Software Version 1.52a was used for image processing.

### Statistical analysis

Statistical analysis was performed using PAST software (Natural History Museum, Norway). Mean values are provided as the mean±s.d. The Kruskal–Wallis H-test followed by a Dunn's test was used for comparisons between multiple groups. Statistical significance: *P<0.05, **P<0.01, ***P<0.001. Details for sample size and replicates used for quantification and the statistical tests applied to determine significance are provided in the figure legends.

## First Person

This article has an associated First Person interview with the first author of the paper.

## References

[DMM050223C1] Abid, Q., Best Rocha, A., Larsen, C. P., Schulert, G., Marsh, R., Yasin, S., Patty-Resk, C., Valentini, R. P., Adams, M. and Baracco, R. (2020). APOL1-associated collapsing focal segmental glomerulosclerosis in a patient with Stimulator of Interferon Genes (STING)-Associated Vasculopathy With Onset in Infancy (SAVI). *Am. J. Kidney Dis.* 75, 287-290. 10.1053/j.ajkd.2019.07.010 31601430 PMC7115721

[DMM050223C2] Aghajan, M., Booten, S. L., Althage, M., Hart, C. E., Ericsson, A., Maxvall, I., Ochaba, J., Menschik-Lundin, A., Hartleib, J., Kuntz, S. et al. (2019). Antisense oligonucleotide treatment ameliorates IFN-γ-induced proteinuria in APOL1-transgenic mice. *JCI Insight* 4, e126124. 10.1172/jci.insight.126124 31217349 PMC6629101

[DMM050223C3] Beckerman, P., Bi-Karchin, J., Park, A. S. D., Qiu, C., Dummer, P. D., Soomro, I., Boustany-Kari, C. M., Pullen, S. S., Miner, J. H., Hu, C.-A. A. et al. (2017). Transgenic expression of human APOL1 risk variants in podocytes induces kidney disease in mice. *Nat. Med.* 23, 429-438. 10.1038/nm.4287 28218918 PMC5603285

[DMM050223C38] Beckerman, P. and Susztak, K. (2018). APOL1: The Balance Imposed by Infection, Selection, and Kidney Disease. *Trends Mol. Med.* 24, 682-695. 10.1016/j.molmed.2018.05.008 29886044 PMC6101980

[DMM050223C4] Bruggeman, L. A., Wu, Z., Luo, L., Madhavan, S., Drawz, P. E., Thomas, D. B., Barisoni, L., O'Toole, J. F. and Sedor, J. R. (2019). APOL1-G0 protects podocytes in a mouse model of HIV-associated nephropathy. *PLoS ONE* 14, e0224408. 10.1371/journal.pone.0224408 31661509 PMC6818796

[DMM050223C5] Cooper, A., Ilboudo, H., Alibu, V. P., Ravel, S., Enyaru, J., Weir, W., Noyes, H., Capewell, P., Camara, M., Milet, J. et al. (2017). APOL1 renal risk variants have contrasting resistance and susceptibility associations with African trypanosomiasis. *eLife* 6, e25461. 10.7554/eLife.25461 28537557 PMC5495568

[DMM050223C6] Friedman, D. J. and Pollak, M. R. (2020). APOL1 and kidney disease: from genetics to biology. *Annu. Rev. Physiol.* 82, 323-342. 10.1146/annurev-physiol-021119-034345 31710572

[DMM050223C7] Fu, Y., Zhu, J.-Y., Richman, A., Zhang, Y., Xie, X., Das, J. R., Li, J., Ray, P. E. and Han, Z. (2017a). APOL1-G1 in nephrocytes induces hypertrophy and accelerates cell death. *J. Am. Soc. Nephrol.* 28, 1106-1116. 10.1681/ASN.2016050550 27864430 PMC5373456

[DMM050223C8] Fu, Y., Zhu, J.-Y., Zhang, F., Richman, A., Zhao, Z. and Han, Z. (2017b). Comprehensive functional analysis of Rab GTPases in Drosophila nephrocytes. *Cell Tissue Res.* 368, 615-627. 10.1007/s00441-017-2575-2 28180992 PMC5429992

[DMM050223C9] Garg, P. (2018). A review of podocyte biology. *Am. J. Nephrol.* 47 Suppl. 1, 3-13. 10.1159/000481633 29852492

[DMM050223C10] Genovese, G., Friedman, D. J., Ross, M. D., Lecordier, L., Uzureau, P., Freedman, B. I., Bowden, D. W., Langefeld, C. D., Oleksyk, T. K., Uscinski Knob, A. L. et al. (2010). Association of trypanolytic ApoL1 variants with kidney disease in African Americans. *Science* 329, 841-845. 10.1126/science.1193032 20647424 PMC2980843

[DMM050223C11] Gerstner, L., Chen, M., Kampf, L. L., Milosavljevic, J., Lang, K., Schneider, R., Hildebrandt, F., Helmstädter, M., Walz, G. and Hermle, T. (2022). Inhibition of endoplasmic reticulum stress signaling rescues cytotoxicity of human apolipoprotein-L1 risk variants in *Drosophila*. *Kidney Int.* 101, 1216-1231. 10.1016/j.kint.2021.12.031 35120995 PMC10061223

[DMM050223C12] Granado, D., Müller, D., Krausel, V., Kruzel-Davila, E., Schuberth, C., Eschborn, M., Wedlich-Söldner, R., Skorecki, K., Pavenstädt, H., Michgehl, U. et al. (2017). Intracellular APOL1 risk variants cause cytotoxicity accompanied by energy depletion. *J. Am. Soc. Nephrol.* 28, 3227-3238. 10.1681/ASN.2016111220 28696248 PMC5661279

[DMM050223C34] Han, Z. and Olson, E. N. (2005). *Hand* is a direct target of Tinman and GATA factors during *Drosophila* cardiogenesis and hematopoiesis. *Development*. 132, 3525-3536. 10.1242/dev.01899 15975941

[DMM050223C13] Hoy, W. E., Hughson, M. D., Kopp, J. B., Mott, S. A., Bertram, J. F. and Winkler, C. A. (2015). APOL1 risk alleles are associated with exaggerated age-related changes in glomerular number and volume in African-American adults: an autopsy study. *J. Am. Soc. Nephrol.* 26, 3179-3189. 10.1681/ASN.2014080768 26038529 PMC4657832

[DMM050223C35] Huang, X., Fu, Y., Lee, H., Zhao, Y., Yang, W., van de Leemput, J. and Han, Z. (2023). Single-cell profiling of the developing embryonic heart in *Drosophila*. *Development*. 150, 10.1242/dev.201936 PMC10482008 37526610

[DMM050223C40] Kimbrell, D. A., Hice, C., Bolduc, C., Kleinhesselink, K. and Beckingham, K. (2002). The *Dorothy* enhancer has Tinman binding sites and drives *hopscotch*-induced tumor formation. *Genesis* 34, 23-28. 10.1002/gene.10134 12324942

[DMM050223C14] Kopp, J. B., Nelson, G. W., Sampath, K., Johnson, R. C., Genovese, G., An, P., Friedman, D., Briggs, W., Dart, R., Korbet, S. et al. (2011). APOL1 genetic variants in focal segmental glomerulosclerosis and HIV-associated nephropathy. *J. Am. Soc. Nephrol.* 22, 2129-2137. 10.1681/ASN.2011040388 21997394 PMC3231787

[DMM050223C15] Kormann, R., Jannot, A.-S., Narjoz, C., Ribeil, J.-A., Manceau, S., Delville, M., Joste, V., Prié, D., Pouchot, J., Thervet, E. et al. (2017). Roles of APOL1 G1 and G2 variants in sickle cell disease patients: kidney is the main target. *Br. J. Haematol.* 179, 323-335. 10.1111/bjh.14842 28699644

[DMM050223C16] Kruzel-Davila, E., Shemer, R., Ofir, A., Bavli-Kertselli, I., Darlyuk-Saadon, I., Oren-Giladi, P., Wasser, W. G., Magen, D., Zaknoun, E., Schuldiner, M. et al. (2017). APOL1-mediated cell injury involves disruption of conserved trafficking processes. *J. Am. Soc. Nephrol.* 28, 1117-1130. 10.1681/ASN.2016050546 27864431 PMC5373454

[DMM050223C17] Lannon, H., Shah, S. S., Dias, L., Blackler, D., Alper, S. L., Pollak, M. R. and Friedman, D. J. (2019). Apolipoprotein L1 (APOL1) risk variant toxicity depends on the haplotype background. *Kidney Int.* 96, 1303-1307. 10.1016/j.kint.2019.07.010 31611067 PMC6907738

[DMM050223C18] Limou, S., Nelson, G. W., Kopp, J. B. and Winkler, C. A. (2014). APOL1 kidney risk alleles: Population genetics and disease associations. *Adv. Chronic Kidney Dis.* 21, 426-433. 10.1053/j.ackd.2014.06.005 25168832 PMC4157456

[DMM050223C19] McCarthy, G. M., Blasio, A., Donovan, O. G., Schaller, L. B., Bock-Hughes, A., Magraner, J. M., Suh, J. H., Tattersfield, C. F., Stillman, I. E., Shah, S. S. et al. (2021). Recessive, gain-of-function toxicity in an APOL1 BAC transgenic mouse model mirrors human APOL1 kidney disease. *Dis. Model. Mech.* 14, dmm048952. 10.1242/dmm.048952 34350953 PMC8353097

[DMM050223C36] Molina-Portela, M. P., Samanovic, M. and Raper, J. (2008). Distinct roles of apolipoprotein components within the trypanosome lytic factor complex revealed in a novel transgenic mouse model. *J. Exp. Med.* 205, 1721-1728. 10.1084/jem.20071463 18606856 PMC2525602

[DMM050223C20] Nichols, B., Jog, P., Lee, J. H., Blackler, D., Wilmot, M., D'agati, V., Markowitz, G., Kopp, J. B., Alper, S. L., Pollak, M. R. et al. (2015). Innate immunity pathways regulate the nephropathy gene Apolipoprotein L1. *Kidney Int.* 87, 332-342. 10.1038/ki.2014.270 25100047 PMC4312530

[DMM050223C21] Okamoto, K., Rausch, J. W., Wakashin, H., Fu, Y., Chung, J.-Y., Dummer, P. D., Shin, M. K., Chandra, P., Suzuki, K., Shrivastav, S. et al. (2018). APOL1 risk allele RNA contributes to renal toxicity by activating protein kinase R. *Commun. Biol.* 1, 188. 10.1038/s42003-018-0188-2 30417125 PMC6220249

[DMM050223C22] Ryu, J.-H., Ge, M., Merscher, S., Rosenberg, A. Z., Desante, M., Roshanravan, H., Okamoto, K., Shin, M. K., Hoek, M., Fornoni, A. et al. (2019). APOL1 renal risk variants promote cholesterol accumulation in tissues and cultured macrophages from APOL1 transgenic mice. *PLoS ONE* 14, e0211559. 10.1371/journal.pone.0211559 30998685 PMC6472726

[DMM050223C23] Takahashi, S., Kubo, K., Waguri, S., Yabashi, A., Shin, H.-W., Katoh, Y. and Nakayama, K. (2012). Rab11 regulates exocytosis of recycling vesicles at the plasma membrane. *J. Cell Sci.* 125, 4049-4057. 10.1242/jcs.102913 22685325

[DMM050223C24] Thomson, R., Genovese, G., Canon, C., Kovacsics, D., Higgins, M. K., Carrington, M., Winkler, C. A., Kopp, J., Rotimi, C., Adeyemo, A. et al. (2014). Evolution of the primate trypanolytic factor APOL1. *Proc. Natl. Acad. Sci. USA* 111, 10.1073/pnas.1400699111 PMC4034216 24808134

[DMM050223C25] Tzur, S., Rosset, S., Shemer, R., Yudkovsky, G., Selig, S., Tarekegn, A., Bekele, E., Bradman, N., Wasser, W. G., Behar, D. M. et al. (2010). Missense mutations in the APOL1 gene are highly associated with end stage kidney disease risk previously attributed to the MYH9 gene. *Hum. Genet.* 128, 345-350. 10.1007/s00439-010-0861-0 20635188 PMC2921485

[DMM050223C26] Tzur, S., Rosset, S., Skorecki, K. and Wasser, W. G. (2012). APOL1 allelic variants are associated with lower age of dialysis initiation and thereby increased dialysis vintage in African and Hispanic Americans with non-diabetic end-stage kidney disease. *Nephrol. Dial. Transplant.* 27, 1498-1505. 10.1093/ndt/gfr796 22357707

[DMM050223C27] Ulasi, I. I., Tzur, S., Wasser, W. G., Shemer, R., Kruzel, E., Feigin, E., Ijoma, C. K., Onodugo, O. D., Okoye, J. U., Arodiwe, E. B. et al. (2013). High population frequencies of APOL1 risk variants are associated with increased prevalence of non-diabetic chronic kidney disease in the Igbo people from south-eastern Nigeria. *Nephron. Clin. Pract.* 123, 123-128. 10.1159/000353223 23860441

[DMM050223C28] Vanhamme, L., Paturiaux-Hanocq, F., Poelvoorde, P., Nolan, D. P., Lins, L., Van Den Abbeele, J., Pays, A., Tebabi, P., Van Xong, H., Jacquet, A. et al. (2003). Apolipoprotein L-I is the trypanosome lytic factor of human serum. *Nature* 422, 83-87. 10.1038/nature01461 12621437

[DMM050223C29] Wakashin, H., Heymann, J., Roshanravan, H., Daneshpajouhnejad, P., Rosenberg, A., Shin, M. K., Hoek, M. and Kopp, J. B. (2020). APOL1 renal risk variants exacerbate podocyte injury by increasing inflammatory stress. *BMC Nephrol.* 21, 371. 10.1186/s12882-020-01995-3 32854642 PMC7450955

[DMM050223C30] Wang, L., Wen, P., Van De Leemput, J., Zhao, Z. and Han, Z. (2021). Slit diaphragm maintenance requires dynamic clathrin-mediated endocytosis facilitated by AP-2, Lap, Aux and Hsc70-4 in nephrocytes. *Cell Biosci.* 11, 83. 10.1186/s13578-021-00595-4 33975644 PMC8111712

[DMM050223C31] Wen, P., Zhang, F., Fu, Y., Zhu, J.-Y. and Han, Z. (2020). Exocyst Genes Are Essential for Recycling Membrane Proteins and Maintaining Slit Diaphragm in *Drosophila* Nephrocytes. *J. Am. Soc. Nephrol.* 31, 1024-1034. 10.1681/ASN.2019060591 32238475 PMC7217423

[DMM050223C32] Xie, X., Colberg-Poley, A. M., Das, J. R., Li, J., Zhang, A., Tang, P., Jerebtsova, M., Gutkind, J. S. and Ray, P. E. (2014). The basic domain of HIV-tat transactivating protein is essential for its targeting to lipid rafts and regulating fibroblast growth factor-2 signaling in podocytes isolated from children with HIV-1-associated nephropathy. *J. Am. Soc. Nephrol.* 25, 1800-1813. 10.1681/ASN.2013070710 24578133 PMC4116058

[DMM050223C33] Zhang, F., Zhao, Y. and Han, Z. (2013). An in vivo functional analysis system for renal gene discovery in *Drosophila* pericardial nephrocytes. *J. Am. Soc. Nephrol.* 24, 191-197. 10.1681/ASN.2012080769 23291470 PMC3559487

